# Mapping Determinants of Cytokine Signaling via Protein Engineering

**DOI:** 10.3389/fimmu.2018.02143

**Published:** 2018-09-27

**Authors:** Claire Gorby, Jonathan Martinez-Fabregas, Stephan Wilmes, Ignacio Moraga

**Affiliations:** Division of Cell Signaling and Immunology, School of Life Sciences, University of Dundee, Dundee, United Kingdom

**Keywords:** cytokine signaling, protein engineering, JAK/STAT signaling pathway, endosomal trafficking, endosomes signaling

## Abstract

Cytokines comprise a large family of secreted ligands that are critical for the regulation of immune homeostasis. Cytokines initiate signaling via dimerization or oligomerization of the cognate receptor subunits, triggering the activation of the Janus Kinases (JAKs)/ signal transducer and activator of transcription (STATs) pathway and the induction of specific gene expression programs and bioactivities. Deregulation of cytokines or their downstream signaling pathways are at the root of many human disorders including autoimmunity and cancer. Identifying and understanding the mechanistic principles that govern cytokine signaling will, therefore, be highly important in order to harness the therapeutic potential of cytokines. In this review, we will analyze how biophysical (ligand-receptor binding geometry and affinity) and cellular (receptor trafficking and intracellular abundance of signaling molecules) parameters shape the cytokine signalosome and cytokine functional pleiotropy; from the initial cytokine binding to its receptor to the degradation of the cytokine receptor complex in the proteasome and/or lysosome. We will also discuss how combining advanced protein engineering with detailed signaling and functional studies has opened promising avenues to tackle complex questions in the cytokine signaling field.

## Introduction

Cytokines comprise a large family of soluble factors, which control virtually every aspect of mammalian physiology (1–5). Deregulation of cytokines or cytokine-related pathways can result in human diseases such as asthma, severe combined immunodeficiency (SCID) and certain cancers (6–13), making this family highly relevant to human health. A poor mechanistic understanding of how cytokine signaling is initiated and regulated in space and time, however, has hindered the translation of these ligands to the clinic.

The cytokine signaling paradigm encompasses the binding of a cytokine to its surface receptors, followed by the activation of receptor associated tyrosine kinases of the Janus Kinases family (JAKs) (1, 14). JAKs in turn phosphorylate tyrosines in the cytokine receptor intracellular domains (ICD), generating docking sites for the signal transducer and activator of transcription (STAT) factors (2, 3, 15, 16). Upon receptor binding, STATs are phosphorylated by JAKs, forming homo- and hetero-dimers, which translocate to the nucleus, bind specific promoter sequences and induce defined gene expression programs and bioactivities (17–19). In recent years however, a series of biophysical and protein engineering studies have provided new evidence which highlights the large complexity of signaling triggered by a cytokine-cytokine receptor complex. This complexity allows cytokines to produce a wide range of biological responses despite using a very minimal set of surface receptors and effector signaling molecules. In this review, we will focus on cytokines that engage the JAK/STAT signaling pathway and on how the engineering of agonistic surrogate cytokines has expanded our understanding of cytokine signaling and biology; in addition we will discuss future directions in the context of cytokine-based therapies.

## Stoichiometry of the cytokine-cytokine receptor complex in the plasma membrane

One of the most debated questions in the cytokine field concerns the stoichiometry of the cytokine receptor complex in the absence of ligand (20, 21). At first glance, this question seems to be unimportant, given that all models agree that ligand binding is the initial step for activating cytokine receptors. However, how cytokine receptors are activated by cytokine binding has clear functional implications—in particular for targeted engineering of desired cytokine properties. Two opposing models have emerged in the past years. The first model postulates that cytokine receptors exist as preformed inactive dimers in the plasma membrane that become active upon cytokine binding through a conformational/structural rearrangement. Evidence supporting this model is found primarily in homo-dimeric systems such as erythropoietin (Epo) (22–26), thrombopoietin (Tpo) (27, 28), and Growth Hormone (GH) (29–31), although some reports in hetero-dimeric systems have also being reported (32–35). The erythropoietin receptor (EpoR) was found to exist as a dimer in crystals that did not include Epo (23), and at the cell surface by immunofluorescence (26). Similar observations were made for the GH receptor (GH-R) via co-immunoprecipitation of differentially tagged receptors or fluorescence and bioluminescence resonance energy transfer techniques (29, 30).

A second model postulates that cytokine receptors diffuse freely in the plasma membrane as monomers and only upon cytokine binding are recruited into a complex to trigger signaling. According to this model, cytokine receptor assembly is driven by affinities, interaction rate constants and the respective concentrations of all involved reactants. This leads to dynamic equilibria between monomeric and assembled receptors subunits, which can be tuned by affinities and receptor concentrations according to the law of mass action. There are also several lines of evidence supporting this model: (a) this model predicts a step-wise formation of cytokine receptor complexes. Indeed, all cytokines described to date bind one of the receptor chains with significantly higher affinity than the other one and step-wise complex formation has been shown for several cytokines both in *in vitro* and *in vivo* studies, including Epo and IFN systems (36–38). (b) cytokine receptor chimeras where the extracellular domains and/or transmembrane domains are swapped by those of any other receptor still trigger signaling in a ligand dependent manner (39–45). (c) surrogate cytokine ligands, e.g., antibodies (46–49) can trigger signaling, arguing against precise conformational changes required for signal activation. (d) Single particle fluorescence imaging studies in several cytokine systems has shown that receptor subunits exist as monomers on the surface of live cells at physiologically relevant cell surface densities, and only form dimers upon ligand binding (48, 50–55). Additionally, cytokines with mutations in the low-affinity chain binding site (“site 2”) fail to induce receptor dimers in agreement with the classical two-step binding mode.

A point to consider from all these studies is that in many instances modified/tagged receptor constructs that are ectopically expressed are used. Thus, the possibility that these modifications inhibit or induce receptor assembly on their own cannot be formally excluded, making it difficult to decide which model is true for a given cytokine receptor system. Nonetheless, due to the strong evidences supporting either model, it is plausible that both models are correct to some extent and that their relative contribution to cytokine signaling could vary depending on cellular context, i.e., receptor and signaling molecules abundance, as has been reported for the Epo system.

## Cytokine-cytokine receptor complex stability vs. activity

A key factor contributing to signaling and bioactivity potency and specificity by cytokines is the stability of the cytokine-cytokine receptor interactions. Type I Interferons (IFNs) have been used as a model system to study how receptor complex stability influences signaling. The type I IFN family comprises more than 15 different subtypes, all binding to the same receptor complex formed by IFNAR1 and IFNAR2 subunits and activating to the same extent the same JAK/STAT pathway (36, 56–58). Yet, different IFN subtypes induce anti-viral and anti-cancer responses with very different potencies (59–64). While all IFNs exhibit a comparable antiviral activity, only IFNβ has an exceptional antiproliferative activity, which is linked to its anti-cancer potential. A series of biophysical, structural and engineering studies has started to address this apparent lack of correlation between signaling and activity output in this family. Early studies elegantly showed that complex stability critically contributed to the differential activities exhibited by IFNs (59, 65–69). Indeed, an IFNα2 variant, engineered to mimic the properties of IFNβ by enhancing IFNAR1 binding affinity acquired potent antiproliferative activity (59, 66, 67). More recently, structural and engineering studies have shown that the topologies of the IFN receptor complexes formed by the different IFNs are very similar and that their differential activities likely result from different receptor binding kinetics and signal activation (64, 70). Indeed, this differential kinetics of STAT activation by type I IFNs result in the induction of two sets of genes: robust genes that drive the antiviral response and only require short pulses of IFN at low concentrations, and tuneable genes that require sustained activation with higher doses of IFNs and are linked to the anti-cancer responses. Induction of robust genes is not very sensitive to changes in complex stability, while the induction of tuneable genes is (71–73).

The ability of cytokine receptors to translate binding stability into biological output potency is not restricted to type I IFNs and can be found in other cytokine systems. IL-4 and IL-13 are two important immunomodulatory cytokines that bind the same receptor complex comprised of IL-4Rα and IL-13Rα1 and activate STAT6 (8, 74). Yet, their activities are not completely overlapping, with each cytokine exhibiting pockets of specificity (75–77). Biophysical and structural studies have shown that the kinetics of complex formation by these two cytokines is at the root of their differences (5, 78, 79). While IL-4 binds first IL-4Rα with high affinity and then recruits IL-13Rα1, IL-13 first binds IL-13Rα1 and then recruits IL-4Rα. Importantly, in most immune cells IL-4Rα appears to be the limiting factor (78, 80–83). As a consequence, IL-4 by recruiting IL-4Rα with higher efficiency can activate signaling more efficiently than IL-13 and overall elicit more potent biological responses (77). Despite this, IL-13 can elicit specific biological responses not induced by IL-4, for which we still lack a mechanistic understanding.

Viruses have taken advantage of the functional plasticity exhibited by the cytokine system. Viruses often encode open-reading frames that share sequence identity with known human cytokines and mimic their biological properties (56, 84–86). A classic example of this is viral IL-10 (vIL-10) (87). IL-10 is a key immune-modulatory cytokine that controls the extent and potency of the immune response by engaging a surface receptor formed by IL-10Rα and IL-10Rβ receptor subunits to activate STAT3 (11, 56, 88, 89). Interestingly two viruses, cytomegalovirus (CMV) and Epstein-Barr (EBV), encode in their genomes homologs of this cytokine (87). Of particular interest is the ebvIL-10, which it is better known as vIL-10. Despite sharing a high degree of sequence and structural homology with human IL-10 (hIL-10), vIL-10 only engages the anti-inflammatory responses elicited by hIL-10 (90–92). vIL-10 inhibits the expression of MHC class II in monocytes and macrophages and the proliferation of T cells (93), but fails to promote other hIL-10 activities such as induction of thymocytes and mast cell proliferation or upregulation of MHC class II by B cells (94–96). This differential effect could be traced to the different complex stabilities elicited by the two ligands, with vIL-10 binding more weakly to IL-10Rα than hIL-10 (91). Overall, these studies describe an intricate relationship between ligand-receptor complex stability and signaling and biological outcomes by cytokines, which could act as a source of functional heterogeneity and potentially be exploited for therapeutic purposes.

## Additional factors contributing to cytokine-cytokine receptor complex stability

**Figure 1:**
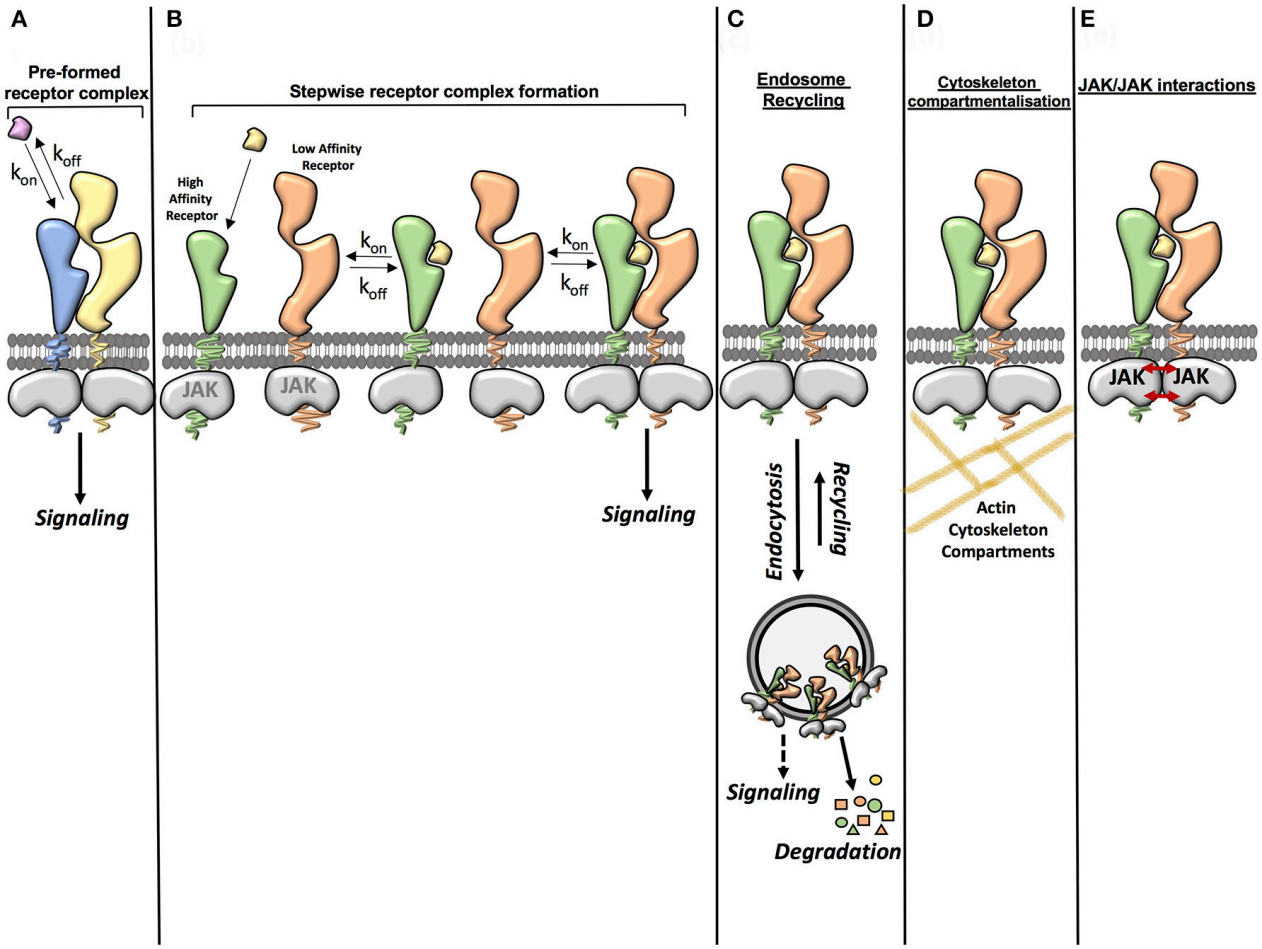
Factors contributing to cytokine-cytokine receptor complex stability. **(A)** Ligand affinity for the receptor (pre-formed receptor complex). The affinity of the ligand for the pre-formed receptor can enhance the stability of the ligand-receptor complex. **(B)** Ligand affinity for the receptor (stepwise receptor complex formation). The ligand first binds to the high affinity receptor chain. This then allows recruitment of the second receptor chain. The affinity of the ligand for the high affinity receptor chain and the affinity of the ligand-high affinity receptor complex for the second receptor chain can influence the stability of the complete complex. **(C)** Endosome recycling. Endocytosis of the ligand-receptor complex from the cell surface to intracellular vesicles depletes ligands and receptors from the plasma membrane, negatively regulating signaling. However some studies have suggested that there is a possibility that some ligand-receptor complexes may also be stabilized in the endosome, leading to a stable complex being formed. Under certain circumstances the recycling of receptor components and ligands from the endosome back to the plasma membrane also contributes to the formation of a stable complex. **(D)** Cytoskeleton compartmentalization. Cytoskeleton components such as actin can contribute to the stability of a receptor complex by confining the movement of the receptor and thus increasing the opportunity for rebinding of the ligand. **(E)** JAK/JAK interactions. Some studies have shown that interactions between JAKs can aid dimerization of the receptor complex and that JAKs can act in trans in certain receptor systems.

Signal activation by cytokines is a very efficient process where cytokines exhibiting a wide range of binding affinities activate signaling to a similar extent. This suggests that other factors beyond the sole affinity of the ligand for its receptor contribute to form and stabilize the cytokine receptor complex (Figure 1). Here we will highlight three cellular determinants that have been the focus of attention in recent studies:

The role of the endosomal compartment in cytokine signaling initiation and diversification has been proposed but not formally proven (97–102). Early work in the EGF system showed that EGF mutants with impaired EGFR binding affinity paradoxically elicited more potent signaling responses (103). Through a series of studies the authors showed that receptor complexes formed by these mutants did not survive the endosomal acidic pH leading to dissociation and recycling of the ligands and receptor to the membrane, contributing to more sustained signal activation by these mutants (103). More recently, studies utilizing TIRF microscopy have revealed that the endosomal compartment contributes to the formation and stabilization of the cytokine-cytokine receptor complex, thus ensuring signaling fitness at a wide range of environmental conditions (50, 104). Whether endosomes serve as signaling platforms where cytokine receptors encounter alternative signaling molecules to fine-tune their activities, however, still remains an open question. Some evidence of this can be found in a study which showed that phosphorylated JAK1 and Tyk2 could be found in EEA1 positive endosomes upon IFN stimulation (105). Additionally, mutations in the G-CSF receptor that altered its intracellular traffic differentially affected the signaling output and bioactivities engaged by this receptor (106, 107). However, to date no direct evidence demonstrating that endosomes function as signaling hubs for cytokine receptors has been described. This dearth of knowledge originates from the technical challenge that following and blocking cytokine receptor complexes to intracellular compartments represents. Future studies combining biochemistry and imaging methodologies will be required to address this long-standing question.

Another factor contributing to cytokine-cytokine receptor complex formation and stabilization is the actin cytoskeleton. Two recent studies in the IFN and IL-4 systems have shown that cytokine receptors are confined to cytoskeletal microcompartments at the plasma membrane, which allows quick reassembly of the cytokine-cytokine receptor complex after dissociation (52, 55). Manipulation of these actin compartments with small molecule inhibitors altered signaling downstream of the IFN or IL-4 complexes (52, 55).

Yet another factor contributing to cytokine-cytokine receptor complex stability are the JAK kinases associated to the receptors intracellular domains. Early studies with type I IFN showed that mutations in JAKs which did not affect receptor surface expression decreased the number of high affinity IFN binding sites in cells, suggesting an inside-out communication between JAKs and the IFN receptor (108). More recent studies have confirmed this initial observation and provided mechanistic insight into this JAK-receptor communication. A first study showed that two JAK2 molecules could interact *in trans* via their kinase/pseudokinase domains when bound to the GH-R homodimer, contributing to signaling initiation and propagation (31, 109). A follow up study showed that a productive JAK1-Tyk2 interaction was required to obtain maximal dimerization efficiency in the IFN system. Indeed, lack of JAK1 resulted in a reduction of the number of complexes formed by IFNα2, which could not be assigned to lower levels of IFNAR1 or IFNAR2 on the surface (51).

Overall these studies suggest that the cytokine system has developed a series of check points to ensure that the cytokine-cytokine receptor complex is formed and activates signaling. The next topic we will address is whether we can exploit these different factors contributing to cytokine receptor complex formation to fine-tune cytokine signaling and responses.

## Exploiting cytokine engineering to discover new cytokine biology

Manipulation of cytokine binding properties via protein engineering is a valuable tool with which we can better understand cytokine biology and to fine-tune cytokine responses. Above we have already introduced some examples focused on the IFN system that help to better understand IFN biology. Next, we will describe additional examples in other cytokine systems which highlight the potential of cytokine engineering to address complex biological problems.

IL-2 plays a critical role in regulating T cell responses, making it an attractive target to treat autoimmune diseases and cancer (5, 110–113). However, its use in the clinic is limited due to severe toxicity resulting from its functional pleiotropy (114–117). IL-2 can engage two types of receptor complexes on the surface of responsive cells: the high affinity receptor complex comprised of IL-2Rα, IL-2Rβ, and γc receptor subunits and the intermediate affinity complex formed by IL-2Rβ and γc subunits (5). Thus, T cells control their sensitivity to IL-2 by modulating their levels of the alpha receptor (113). Many attempts to improve the clinical efficacy of IL-2 by fine tuning its receptor binding properties have been carried out over the years. One of the first studies was performed by Shanafelt and colleagues, who proposed that IL-2-derived toxicity resulted from engagement of the intermediate receptor present on NK cells, which are believed to be the major source of the cytokines and inflammatory mediators causing most of the toxicity associated with high-dose IL-2 therapy (118). In order to specifically target IL-2 to T cells and thus decrease its toxicity they used site directed mutagenesis to reduce the binding affinity of IL-2 to IL-2Rβ (119). This IL-2 mutant could not engage the intermediate affinity receptor, but still could activate signaling in the context of the high affinity receptor, leading to a more than 3,000-fold specificity for T cells over NK cells (119). In an experimental lung metastasis model, sensitive to IL-2 therapy, this IL-2 mutant showed similar levels of tumor inhibition to IL-2 but elicited lower levels of morbidity as scored by general health examination (119). However, in a later phase I trial this mutant did not show advantage over wt IL-2 in anti-tumor responses or toxicity, highlighting the complexity of this cytokine in an *in vivo* setting (120). Another example of IL-2 engineering is found in studies by the Wittrup lab. Using yeast surface display, the authors engineered an IL-2 variant with high affinity for IL-2Rα. This variant induced T cell proliferation more potently than wt IL-2, suggesting that it could be a better alternative than wt for cancer immunotherapy since lower doses of the variant would be required to show efficacy which could result in lower toxicity (121–123).

More recently, studies by Garcia and collaborators have provided a series of IL-2 variants that have furthered our understanding of IL-2 biology. Using yeast surface display, Levin and colleagues engineered an IL-2 variant (Super-2) binding 200-fold tighter to IL-2Rβ than wt (124). Super-2 can signal through the intermediate affinity receptor as potently as through the high affinity receptor, thus negating the regulatory role of the alpha subunit. This in turn resulted in a stronger anti-tumor response by Super-2 with a significantly lower toxicity when compared to wt IL-2 (124). In a second study, Suman and colleagues used Super-2 as a backbone to engineer a series of Super-2-based cytokines where binding to γc chain was reduced (125). Strikingly, the authors observed that rather than a complete loss in response, these new variants activated signaling with different amplitudes ranging from 100% activity to 50 and 10% in accordance with their binding affinity (125). Interestingly, the IL-2 mutant activating 50% activity could induce proliferation of activated T cells, but not of naïve T cells, suggesting different signaling thresholds required for proliferation in different T cell differentiation stages (125).

In addition, a recent study has shown that IL-2 receptor binding specificity can also be altered in a mutation-independent manner by introducing PEG molecules in the IL-2 region interacting with IL-2Rα. This new IL-2 variant, named NKTR-214, has shown promising anti-tumor responses and decreased toxicity and it is now finding its way to the clinic (126).

The IL-4/IL-13 system has also been the subject of protein engineering studies. As described above, IL-4 binds two surface receptor complexes: The type I receptor, consisting of the IL-4Rα and γc subunits, which is found exclusively on hematopoietic cells; and the type II receptor, composed of the IL-4Rα and IL-13Rα1 chains, which is also shared by IL-13 (5, 78). A recent work by Junttila and collaborators shed some light onto the differential activities elicited by the two IL-4 complexes. Using yeast surface display, the authors engineered two IL-4 variants exhibiting high specificity for either the type I or the type II IL-4 receptors (127). Detailed functional characterization of these variants revealed that while T cell responses were exclusively dependent on the type I IL-4 complex, in agreement with the specific expression of this receptor in T cells, dendritic cell maturation was dependent on the IL-4 type II complex (127). These results agreed with previously published observations and revealed functional dichotomy between the Type I and Type II IL-4 receptors (128, 129).

The impact of complex formation kinetics and stability on signaling and activities by the IL-4/IL-13 complex was further explored in a recent work (50). In this study, we engineered a range of IL-13 variants exhibiting different binding affinities for the IL-13Rα1 receptor subunit. When we functionally characterized these variants, we observed that large decreases in binding affinity were required to marginally alter signaling efficiency. Further increases in binding affinities, however, did not improve signaling by IL-13. Through a series of modeling simulations and experiments we concluded that transition of the cytokine-cytokine receptor complex to the endosomal compartment was the limiting rate factor for signaling potency in the IL-13 system. Cytokine-cytokine receptor complexes capable of undergoing endocytosis would be stabilized due to the high local cytokine receptor concentration achieved in the limited area of endosomes. Further stabilization of the complex beyond that required to transit to the endosomes will have minimal influence on signaling (50). Indeed, our data agreed with a recent study highlighting the role of the endosomal compartment in the formation of the IL-4/IL-13 complexes (104, 130). Interestingly, this disconnect between binding affinity and signaling output was not found when more complex biological responses, e.g., TF-1 cell proliferation and dendritic cell differentiation, were analyzed, which in turn directly correlated with the stability of the IL-13 complex (50). A possible explanation for this apparent lack of correlation between signaling and activity could be found in the different times used to study these processes. While signaling is measured during the first few hours of cytokine stimulation, biological responses often take days to be observed. Surface receptors, signaling molecules and ligand concentrations could be altered with time, leading to functional diversification from an apparently similar starting point. An example of this can be found in the type I IFN system, where IFN stimulation leads to the upregulation of negative regulators that preferentially inhibit short-lived IFN complexes (51, 131, 132).

## Conclusions and remarks

In this mini-review, we have summarized recent studies that have underlined the intricate interplay of cytokine-receptor complex stability and signaling and biological responses. Additionally we have discussed recent findings that support a scaffolding role for the JAK kinases in complex formation, as well as interesting observations regarding the contribution of the actin cytoskeleton and the endosomal compartment to signaling robustness by cytokine-receptor complexes. However, key standing questions remains in the field such as how binding of a cytokine to its receptor triggers signaling, how signaling specificity is generated, are endosomes contributing to fine tune cytokine signaling and biology and how cytokine functional pleiotropy is generated. In order to answer these questions, which would allow us to rationally manipulate cytokine responses and harness their full therapeutic potential, future studies will need to take advantage of recent advances in cryo-EM and membrane protein structural biology to fully understand the complex interconnectivity of the cytokine/cytokine receptor/JAK/STAT complex. Additionally, advance microscopy studies combined with proximity labeling methodologies such as bioID could provide us with new insights into the role that the endosomal compartment plays in shaping cytokine signaling and responses.

## Author contributions

All authors listed have made a substantial, direct and intellectual contribution to the work, and approved it for publication.

### Conflict of interest statement

The authors declare that the research was conducted in the absence of any commercial or financial relationships that could be construed as a potential conflict of interest.
